# Parent and staff focus groups to address NICU racial inequities: “There’s radical optimism in that we’re in a different time and we’re not doing it alone”

**DOI:** 10.1038/s41372-024-02063-6

**Published:** 2024-07-18

**Authors:** Kayla L. Karvonen, Olga Smith, Brittany Chambers-Butcher, Patience Afulani, Tameyah Mathis-Perry, Khuzaima Rangwalla, Monica McLemore, Elizabeth E. Rogers

**Affiliations:** 1 Department of Pediatrics, University of California San Francisco, San Francisco, CA USA; 2 California Preterm Birth Initiative, San Francisco, CA USA; 3 Independent Researcher, Antioch, CA USA; 4 Department of Human Ecology, College of Agricultural and Environmental Sciences, University of California, Davis, Davis, CA USA; 5 Department of Epidemiology and Biostatistics, University of California San Francisco, San Francisco, CA USA; 6 Morehouse School of Medicine, Atlanta, GA USA; 7 University of California San Francisco School of Medicine, San Francisco, CA USA; 8 Department of Child, Family, and Population Health Nursing, University of Washington, Seattle, WA USA

**Keywords:** Paediatrics, Health services

## Abstract

**Objectives:**

To understand local mechanisms of racial inequities and generate recommendations from community members regarding how to promote racial equity in the Neonatal Intensive Care Unit (NICU).

**Methods:**

In an urban tertiary care NICU, 4 semi-structured in-person focus groups with follow-up audio diaries were conducted with NICU parents and staff from 2022–2023 with support from interpreters, a psychologist, and a family advocate. Researchers coded transcripts independently and thematic analysis was utilized to generate and refine themes.

**Results:**

16 racially diverse and multidisciplinary staff and parents participated, and six themes emerged from the data. Mechanisms of racial inequities included power dynamics, interpersonal and institutional dehumanization, and societal inequities. Recommendations included redistributing power, transforming space and staff to promote humanism, and mitigating harm through peer support and resource allocation.

**Conclusion:**

Focus groups are a promising strategy to identify interventions to address racial inequities. Future research should focus on intervention implementation and evaluation.

## Introduction

Racial inequities in health outcomes experienced by racially marginalized individuals are pervasive. Across all life stages, racially marginalized groups are at higher risk of a range of morbidities and mortality, due to downstream impacts of structural racism and resource inequities [1, 2]. The birthing process is no exception. For centuries, Black and Indigenous groups have been at disproportionate risk of complications and mortality during childbirth, and infant mortality and prematurity [3, 4]. After birth, racial inequities have been described in infant comorbidities, health care utilization, and mortality after discharge [5–8]. Families have described their dissatisfaction with the healthcare they have received and racism in healthcare settings throughout the birthing process, from their obstetric to neonatal intensive care unit (NICU) care [9–13]. Despite their recognition, racial inequities in neonatal outcomes persist.

One important strategy to enhance innovation and relevance of research to address racial inequities is to shift discourse from the majority to the marginalized perspective, a core principle drawn from Black feminism and critical race theory called centering in the margins [14, 15]. Community engagement throughout the research cycle is recommended for conducting ethical research, maximizing reach and benefit of findings, disseminating research back to the community, and enhancing community-academic partnerships and trust [16]. In the NICU, several frameworks support a community centered approach to anti-racism that include consultation of a diverse family advisory board and community engagement in clinical and academic settings [8, 17–19]. Other qualitative studies have capitalized on these principles by interviewing family members or staff separately about their experiences in the NICU generally and recommendations for how to improve their experiences [9–12]. There are very few studies that have facilitated discussion of members across roles by including both staff and parents, and across different racial and ethnic backgrounds, to come together to make recommendations for how to address racial inequities locally.

The Racial and Ethnic Justice in Outcomes in Neonatal Intensive Care (REJOICE) study is a mixed methods study that applies these concepts in a local approach to address racial inequities in the neonatal intensive care unit (NICU). Phase one of the REJOICE study identified (1) racial inequities in neonatal health outcomes and adverse social events [20] and (2) parent and staff experiences with racism and discrimination [21, 22]. Electronic medical record, survey, and interview data informed phase 1. In an effort to continue centering in the margins while moving into action, the objective of phase 2 is to understand the perspectives of NICU staff and marginalized parents regarding the mechanisms by which racial inequities identified in phase 1 are perpetuated and to obtain paired consensus recommendations for how to directly address them. Mechanisms and recommendation are explicitly paired to provide action-oriented solutions to the problems identified.

## Methods

### Setting and participants

The study recruited participants from a single-center urban tertiary care center NICU. Eligibility criteria for parents to participate were those who self-identified as Black/African-American (hereafter referred to as “Black”) and Hispanic/Latinx (hereafter referred to as “Latinx”), who had children who had been admitted to the UCSF NICU in the last 3 years, and who spoke English or Spanish as their primary language. Black and Latinx parents were specifically invited as both marginalized groups were most impacted by the local racial inequities found in phase 1 of the REJOICE study and to practice the Black feminism and critical race theory principles of shifting discourse from the majority to the marginalized perspectives [14, 15]. To further embody these principles, racially marginalized researchers, a family advocate, and a psychologist led the study from design to completion. Eligibility for staff members included any staff member who was employed by the UCSF NICU during the study period. Utilizing hospital admission lists, parents were recruited by phone, email, and in-person in the NICU by racially diverse staff members and remotely through study flyers distributed throughout the NICU.

### Procedures

In conference rooms located within the hospital facility network, four separate 4-hour semi-structured focus groups were conducted with Black and Latinx parents and multidisciplinary racially diverse staff members from July 2022–July 2023. In-person focus groups were conducted by three researchers who were employed as medical providers in the NICU, with in-person Spanish interpreters. A psychologist and family advocate also attended sessions for additional parent support and led an empowerment exercise to start the first session. Group size was consistent with thematic saturation recommendations, ranging from 5–9 participants with 2–5 research team members [23]. A brief questionnaire developed by the research team and focus group transcription data was used to collect demographic information. The semi-structured focus group guide allowed for introductions including additional relevant information regarding intersectional identities. Each session started with a presentation by study researchers of a unique portion of the phase 1 REJOICE study findings. For example, session 1 started with a presentation on local quantitative data on racial inequities in treatment, standards of care, outcomes, and adverse social events. The second session presentation covered qualitative survey and interview data from parents. The third session presentation covered staff survey and interview data and no new data was provided in session 4. The content of the focus group guide probed for reactions to the racial inequity data from the REJOICE study, personal experiences of racism and discrimination in the NICU, mechanisms by which these experiences occurred, and recommendations for how to address racial inequities (Supplementary Appendix). Sessions were audio recorded and transcribed by a non-AI based transcription company [24]. Moderators of the sessions facilitated discussion regarding rules for engagement and expectations, as well as gave permission to stop participating or to receive psychosocial support at any time from a mental health provider given the sensitive nature of discussions. Focus groups were specifically designed to acknowledge and disrupt power and hierarchy imbalance of providers and care receivers by explicitly naming power and hierarchies in an introductory empowerment session with parent participants only. Children were invited to the sessions for parent convenience, although one parent’s child was still hospitalized in another unit in the hospital, and two parents experienced hospital readmissions that impacted their attendance. After focus groups, staff and parents were invited to record an optional audio diary about their experience participating in the focus groups after each session. Participants received remuneration for their time, based on local compensation rates, and a 1 hour break with lunch provided were included in the sessions. The University of California San Francisco Institutional Review Board reviewed this study and it was determined to be exempt. All participants provided verbal informed consent to participate.

### Data analysis

Focus groups discussions were audio recorded, transcribed, and uploaded into Dedoose for data management [25]. The analysis was conducted by three members of the research team who functioned as three independent coders. The research coders developed a preliminary code book in anticipation of possible themes conceptualized during the focus group development. Additional inductive codes not originally included were also added as data was analyzed as necessary. After coding each session, the research team met to review excerpts and their codes that were incorporated into a final codebook that included definitions of each code. The three researchers independently coded the transcripts and met after coding each session to reach a consensus on any disagreements of how excerpts were coded. Thematic analysis was used to analyze the data [26]. Themes were generated and refined through comparison and discussion over multiple sessions, ultimately consensus was reached within the study team. Thematic saturation was reached by session 3, in that no new observations were noted, and no further themes were identified. After the analysis, the manuscript was reviewed and participant feedback was given.

## Results

A total of 16 people, including 8 staff and 8 parents, participated across 4 focus groups. Table 1 describes participant demographics. There was similar representation of females (*n* = 9, 56.3%) and males (*n* = 7 43.8%). Participants were racially and ethnically diverse with most participants identifying as Black (*n* = 6, 37.5%) or Latinx (n = 5, 31.3%). Participating staff represented a diverse multidisciplinary group of clinicians and clinician leaders, and family social support staff (*n* = 4, *n* = 4). Most participants attended one session (*n* = 9, 56.3%) although many attended multiple sessions (*n* = 7, 43.8%). Six audio diaries were completed by staff and parents. Six themes emerged from the data, including 3 themes describing mechanisms by which racial inequities occurred (Table 2) and 3 themes of recommendations to address them, summarized below (Table 3). Racial inequities occurred through power dynamics, dehumanization and racism (interpersonal and institutional), and societal inequities. Recommendations included redistributing power by treating parents as true care partners, transforming space and staff to promote humanism and inclusion, and mitigating harm through peer support and resource allocation.

**Table 1:** Focus group participant demographics.

		*n*	%
Participant type			
	Caregiver	8	50.0
	Staff	8	50.0
Gender			
	Female	9	56.3
	Male	7	43.8
Race/ethnicity			
	Latinx	5	31.3
	Black	6	37.5
	White	3	18.8
	Asian	2	12.5
Staff type			
	Clinician or clinician leadership	4	50.0
	Family social support staff	4	50.0
Attendance			
	One session	9	56.3
	More than one session	7	43.8
Total		16	100

**Table 2:** Mechanisms of racial inequities themes and exemplar quotes.

Mechanisms	Exemplar Quotes
Complex power dynamics	“We’re all humans but your hands are instruments of life and death and your mind and the way you communicate with us is either going to put us at peace or at hell.” -Black parent
	“You all got the right to call CPS. You all got the right to call security. You got the right to stop us from coming up here. You all got so many rights.” - Black parent
	“I [hear from] Latina moms feeling like they can’t speak up. They don’t have a voice and they just kind of have to whatever the medical team says. Like just kind of follow their recommendations because they’re the ones that are in power and have authority.” - Staff
	“I did talk a lot about it [unequal care] with my social worker. But also there exists this, like … fear that he would be left alone, and because I complained they are going to neglect him. Or, they are going to treat me like –‘ah, she complains a lot; she doesn’t like what we do.’” – Latinx parent
Interpersonal dehumanization and racism	“I had to be this quiet, humble, speak like you got some sense type person rather when I should have had an open space to express how I felt …So that kind of like, I guess you say scared me straight with the way I was interacting with doctors and nurses and with certain people.” -Black parent
	“When I was in the NICU I was trying hard not to be the angry Black woman because I was like, Dang, if I come up there crazy, then I might not get back up there.” – Black parent
	“On some occasions, with some nurses, they saw me differently because of my language, because perhaps because they didn’t understand me, it complicates things for them. [It’s] not so bad, but, well, one feels it.” - Latinx parent
	“Well, in this area they always take care of two or three babies. And, well … what I have observed, when he is there, it’s that … they – they pay more attention to the American babies, than for me – for my baby.” - Latinx parent
Institutional dehumanization and racism	“But I feel like something that keeps coming to my mind listening to all of this is like the way the healthcare system is set up in this country does not promote human interaction in the healthiest way. Everybody is overworked and everybody is worried about getting to the next thing… And just everybody being so busy all the time…It’s so gigantic and so regulated that it removes a lot of the humanity from it.” - Staff
	“Because as a mom, I wish I had just one nurse to take care of him all the time. The nurses, they tell me, ‘there aren’t many of us nurses,’ they tell me. ‘We have to divide ourselves in three, almost,’ and that sometimes they have to care for three babies at a time.” - Latinx parent
	“But then in reality, what happens is actually the people who already have a lot get the most.” - Staff
Societal inequities exacerbate the process of dehumanization	“I just really want us, as healthcare providers to just really consider what it means for a mom to have to live with such precarity. Just to have to come into this space where you’ve already been through this traumatic experience of giving birth to a baby that even needs to be in the NICU.” - Staff
	“Well, yes, it is very hard. Because you have a baby, that is sick, Right? And it is a lot of stress, it is a lot of problems. You have the other things that you are thinking about, more problems. When I am in the hospital, I have everything, they take care of me… but what am I going to do when I go home? And nobody protects you.” - Latinx parent
	“Because I know with my son, I had rent due, I had Christmas around the corner, I had other stressors that made it harder for me to jump up, get to the hospital and just worry about my son. That could have played a part into my anger. That could have played a part into my sadness. It could have played a part into depression. It could have played a part into postpartum, anything.” - Black parent

**Table 3:** Recommendations to address racial inequities and exemplar quotes.

Recommendations	Exemplar Quotes
Redistribute power by treating families as true care partners through orientation, education, and support rather than disciplinary action.	“So… acknowledge the people that is trying, that is caring, that wants their child to be healthy. Give them parents plans. Give them… goals. ‘Hey, we wanna do this. Six months, this should happen,’ or, ‘three months, where we can be here.’” - Black parent
	“We could create like a peer support person, um…to walk the mother through, within the first 24 to 72 hours” - Staff
	“Rather than giving you the support that you’re longing for. What do we say when a child acts out? They need attention. They just want to be heard. Same thing on an adult when they act out. Something’s lacking. They need something… Let’s get to the root of the problem. Let’s sit this family down. Maybe this family needs some extra support. Maybe this family isn’t being heard.” - Black parent
Transform space and staff to promote humanism and inclusion through diversification of staff, minimum criteria for parent-staff interactions, and reduced workload for staff.	“Every child deserves that extra lift of care. Every child deserves that extra minute of research. Every child deserves that extra thought second of, “maybe this can work.” - Black parent
	“Acknowledge you messed up. Acknowledge you read that paper wrong. Acknowledge you miscalculated those numbers. Acknowledge it because when we find it, and we find an error and you don’t acknowledge it, it makes us feel like you’re just doing your job with no compassion and no care in the world.” - Black parent
	“I think it’ll be nice if they had like little daily... Little daily surveys for each, like… like, NICU room, you know? Let’s go over our hospitality. How are you -- how are you -- feel like you’re being treated? How do you feel like your childcare is going? How do you -- are you -- feel like you’re being heard?” - Black parent
Mitigate harm experienced within and outside of the NICU through peer support and resource allocation (social drivers of health) for families.	Regarding focus groups: “It was good to know that there was a lot of other families that went through the same things that I went through and that I wasn’t crazy. It was just nice to hear. I felt it very therapeutic and kind of relieving. It felt good to listen to other moms talk about their journeys and what they experienced in the ICN. It took me a while to reflect on them [my emotions]. I had a day to myself the day after to kind of work on them just because it brought up a lot of old trauma I guess from the ICN that I hadn’t worked on yet but it helped. It helped a lot.” - Black parent
	“We need an outpatient social worker. Not just an inpatient.” - Latinx parent
	“More resources for our families. Yeah, more support services and also the family advisory council. We need more diversity for sure.” - Black parent
	“I would like to see a psychologist on staff, or multiple psychologists on staff because there is one of me and 60 families so I can’t spend the amount of time in providing support. So at least as many social workers, at least as many psychologists as there are social workers so that families can actually have the space to talk about what is happening and how they struggle.” - Staff

### THEME 1: Mechanism: complex power dynamics

Racial inequities occur through multiple and complex power dynamics between parents and staff. Parents enter the NICU space with little power compared to staff who have both medical knowledge and the ability to initiate disciplinary action. In response, parents engaged in self-advocacy or silence for fear of retribution. Both parents and staff acknowledged power dynamics and hierarchy within the medical system as contributors to racial inequities in healthcare. While all parents stated their general gratitude for healthcare professionals and their care, they also highlighted the power to make medical decisions for hospitalized children that impact their health and wellbeing as inherent to the role. The severity and consequence of the power dynamic was articulated by a Black parent to staff, “We’re all humans but your hands are instruments of life and death and your mind and the way you communicate with us is either going to put us at peace or at hell.” A staff member also acknowledged this shared understanding of an inherent power dynamic in the medical system from their interactions with parents, “I [hear from] Latina moms feeling like they can’t speak up. They don’t have a voice and they just kind of have to whatever the medical team says. Like just kind of follow their recommendations because they’re the ones that are in power and have authority.” In addition to making medical decisions, parents also noted staff member’s power to exercise an extensive range of disciplinary action, “You all got the right to call CPS. You all got the right to call security. You got the right to stop us from coming up here. You all got so many rights.”

Yet another power dynamic often begins as parents first enter the NICU is a medical knowledge differential. As the hospital stay lengthens and medical complications arise, this knowledge gap can widen rather than close, depending on the extent of shared communication. Parents all expressed a strong desire to close the knowledge gap throughout their hospital stay. When one Black parent sensed the knowledge gap widening, she met her medical team with self-advocacy, making her needs clear: “I need to know. I want to be informed. I want to be on top of it. I need your number, your number, your number. I’m going to email you. I’m going to text you. I want to be in it because and for me to be in it I need a team behind me. And I’m working with over 20 people from physical therapy, doctors, specialists, the list goes on. But essentially I need my team because my team is going to help me with my son. At first I didn’t have a team. I was still navigating who was who at rounds, talking to different doctors, putting my plan together and what I felt like my son needed, and I was heard.” Here she redistributes the power imbalance by painting a clear picture of the child’s health and well-being team with herself centered in the middle as the child’s primary parent and the medical providers surrounding her.

Other families were silenced by fear of retribution. One staff member said, “That’s something that I often hear from families. They’ll come to me and they’ll express concerns about the nursing care or not agreeing with the doctors as something was said or done that they were really unhappy about and I’m like, ‘Okay, what can we do? Can we talk to the charge nurse or the [nurse manager]’ and oftentimes a lot of the moms will say, ‘No, I just want to tell you but I don’t want anyone else to know, because … what if they don’t take care of my baby.’” One Latinx parent said, “I did talk a lot about it [unequal care] with my social worker. But also there exists this, like … fear that he would be left alone, and because I complained they are going to neglect him. Or, they are going to treat me like – ‘ah, she complains a lot; she doesn’t like what we do.’”

### THEME 2: Recommendation: redistribute power by treating families as true care partners through orientation, education, and support without disciplinary action

There was unanimous desire among the participants for a complete transfer of knowledge from medical providers to parents throughout the hospital stay regarding their infant’s condition, future milestones, potential complications, and how to care for their complex medical needs at home. This starts with a timely and thorough orientation of the NICU, including what resources and personnel are available and how to access them. Different families may need different modes of orientation and all should be available, from an informational sheet of resources and phone numbers in each hospital room to a video and/or in-person orientation. A Black parent asks that parents be shown compassion and given the benefit of the doubt with a plan and therefore future to hope for, “So… acknowledge the people that is trying, that is caring, that wants their child to be healthy. Give them parents plans. Give them goals. ‘Hey, we wanna do this. In six months, this should happen,’ or, ‘three months, we can be here.’” Parents desired to be true care partners during the hospitalization in anticipation of becoming the primary parents at home. Participants emphasized the importance of training for hospital discharge and a life at home when the hospital staff are no longer there. Families described their efforts navigating complex systems, including outpatient medical and therapy appointments, equipment companies, and nursing support.

Participants also recommended providing added layers of support when a parent presents in crisis and strongly recommended against disciplinary actions. One Black parent imagined what families are experiencing when presented with disciplinary action: “Rather than giving you the support that you’re longing for. What do we say when a child acts out? They need attention. They just want to be heard. Same thing on an adult when they act out. Something’s lacking. They need something … Let’s get to the root of the problem. Let’s sit this family down. Maybe this family needs some extra support. Maybe this family isn’t being heard. Maybe this family is lacking something outside of what’s wrong with the child.” By delivering trauma-informed and responsive care, staff can provide holistic care while supporting the family unit, and avoid restrictions or disciplinary action.

### THEME 3: Mechanism: dehumanization and racism

Dehumanization is the denial of recognizing and/or respecting the humanity of other people, whereas racism is a form of dehumanization that denies the humanity of other people based on their race, ethnicity, or color. Racist dehumanization can be practiced by individual people (interpersonal) or by institutions or systems (institutional) [27].

### Subtheme: interpersonal dehumanization and racism

Parents experienced the NICU as a primarily white space in which they needed to change their behavior to assimilate accordingly. Black and Latinx parents both felt racialized by staff, although the ways in which they were racialized were unique. Black parents described being racialized as incompetent, angry, or difficult rather than competent, inquisitive, or an advocate. Parents described times when they asked thoughtful questions or presented their own research and opinions to staff regarding their child’s care and were met with surprise or praise. The assumption and impact on the parent of the staff’s response was that the parent was “not educated or smart”. In situations of parent advocacy accompanied by a display of their emotion, participants recalled instances where white parents were praised as strong advocates, whereas Black parents were labeled as problematic or difficult. In response, Black parents kept their true identities and feelings close and private, and changed their behavior to assimilate to avoid hyper-surveillance, disciplinary action, or other policing. This extra cognitive burden was additive to those intrinsic to having a hospitalized child: One Black parent likened the feeling of limiting who you are in fear of policing similar to the feeling of “a caged animal”. Another Black parent said, “I had to be this quiet, humble, speak like you got some sense type person when I should have had an open space to express how I felt …So that kind of like, I guess you say scared me straight with the way I was interacting with doctors and nurses.” The fear of retaliation was shared and power dynamics described above are evident here, as described by another Black parent, “When I was in the NICU I was trying hard not to be the angry Black woman because I was like, ‘Dang, if I come up there crazy, then I might not get back up there’”.

Latinx parents described strong gratitude for high quality care throughout the sessions and rarely named racism or the experience of being racialized. However, despite rarely attributing instances to discrimination, they described many instances of suboptimal quality of care. In cases of suboptimal care, they were rendered as invisible or erased, and described instances of staff taking care of their child without greeting the parent, introducing themself, or making eye contact. One Latinx parent said “On some occasions, with some nurses, they saw me differently because of my language, because perhaps because they didn’t understand me, it complicates things for them. [It’s] not so bad, but, well, one feels it.” They recounted several distressing medical errors including a medication administered in error, inadequate administration of pain medications due to a line malfunction, and the loss of parent supplied breastmilk. Language discordance on its own was not identified as a primary barrier to high quality care, as Latinx parents described that compassion can supersede language discordance, evidenced by strong bonds with language discordant primary nurses. At the same time, they recounted experiences where language concordance strengthened interpersonal bonding and facilitated easier communication with staff.

### Subtheme: institutional dehumanization and racism

Healthcare systems are structured in a way that perpetuates dehumanization rather than humanism. Staff collectively acknowledged internal motivation for entering the healthcare profession and their daily motivation: to care for others. Staff surmised that systemic pressures on healthcare professionals to perform to meet demands, including time constraints and task workload, can detract staff from providing humanism. One staff member reflected, “The way the healthcare system is set up in this country does not promote human interaction in the healthiest way. Everybody is overworked and everybody is worried about getting to the next thing… And just everybody being so busy all the time…It’s so gigantic and so regulated that it removes a lot of the humanity from it.”

Parents also perceived staff as understaffed and overworked during their time in the NICU. Throughout the focus group sessions, parents found meaning in recognizing staff as real people who have real emotions working within the system. Furthermore, despite the perceived traditional power dynamics, staff did not always have the power to change a system that may be failing a family. A Latinx parent reflected on the 3:1 patient to nurse ratios for less medically complex infants, “Because as a mom, I wish I had just one nurse to take care of him all the time. The nurses, they tell me, ‘there aren’t many of us nurses, we have to divide ourselves in three, almost,’ and that sometimes they have to care for three babies at a time.” When resources are strained they are diverted to privileged groups, a Latinx parent said “Well, in this area they always take care of two or three babies. And, well … what I have observed, when he is there, it’s that … they – they pay more attention to the American babies, than for me – for my baby.” A staff member agreed, “But then in reality, what happens is actually the people who already have a lot get the most.”

Parents also noted the grief, stress, and trauma that healthcare professionals experience regularly as a part of their job and that removing themselves from the emotion, compartmentalizing, and avoiding reflection can be coping tactics. As experiences were shared, parents learned about the shared emotional impact of caring for their children which parents had not previously deeply considered. In a touching sharing of worry, parents expressed concern for staff working conditions and mental health, as perception of the power dynamics shifted towards the center, “You all do a lot. That’s traumatizing to you all. I never even thought about it until right now what that must be like for you all.”

### THEME 4: Recommendation: transform space and staff to promote humanism and inclusion through diversification of staff, minimum criteria for parent-staff interactions, and reduced workload for staff

Improving humanism was a consistent recommendation identified by participants with the core objective of combating instances of lack of humanism broadly and more specifically avoiding racialized experiences. Mechanisms by which to improve humanism in NICU spaces was to change the demographic composition of the staff, institute minimum criteria for interactions with parents by staff, and to increase humanity towards staff to increase their capacity to show humanism by reducing their workload.

Racial and cultural concordance of staff and parents were identified as potentially protective against racialized experiences. Participants described psychological safety and reduced identity threat when conversing with racially and culturally concordant individuals, although a lack of workforce diversity was noted to be a significant barrier to those experiences. Specifically Black and Latinx parents requested more Black and Latinx staff, and staff who spoke Spanish.

In addition to substantively changing staff demographics, participants recommended setting minimum criteria for basic humanism from staff, including making eye contact, greeting, and introducing themselves when staff enter patient spaces, everyday, for every encounter. Beyond minimum criteria, one Black parent explained showing humanism also requires showing humility: “Acknowledge you messed up. Acknowledge you read that paper wrong. Acknowledge you miscalculated those numbers. Acknowledge it because when we find it, and we find an error and you don’t acknowledge it, it makes us feel like you’re just doing your job with no compassion and no care in the world.” Staff workload was identified as a systemic barrier to humanism; when staff seemed busy, sleep-deprived, or fatigued, with numerous competing tasks for their attention, the amount of time and compassion toward parents was reduced. In a setting as important as healthcare for children, any capacity taken from workers to provide high quality care was deemed unacceptable, one parent articulated, “Every child deserves that extra lift of care. Every child deserves that extra minute of research. Every child deserves that extra thought of, ‘maybe this can work.’”

### THEME 5: Mechanism: societal inequities exacerbate the process of dehumanization

The NICU can be a traumatizing space, which is compounded onto other struggles families may face secondary to structural racism. Throughout the sessions, parents shared experiences of racism and discrimination in other medical settings within the hospital, including labor and delivery and other pediatric floors, as well as in other hospitals prior to and after their NICU hospitalization. Navigating racism in the NICU was not an insular event, but rather an addition to their multitude of everyday experiences with racism. Parents also met significant social challenges outside of the hospital, including but not limited to: immigration agency conflicts, difficulty paying rent and providing childcare for their other children, and housing insecurity. One Black parent said, “Because I know with my son, I had rent due, I had Christmas around the corner, I had other stressors that made it harder for me to jump up, get to the hospital and just worry about my son. That could have played a part into my anger. That could have played a part into my sadness. It could have played a part into depression.”

Parents were grateful to the social work team who helped them navigate several of these challenges while they were hospitalized and emphasized the absence and therefore need for more social work support and services after hospital discharge. For families, social services were life lines when their family’s basic needs were threatened. Importantly, despite social work support and connections to resources, there were insurmountable social barriers and parents did have residual needs that were not met. One Latinx parent described this situation as particularly dire at hospital discharge, “Well, yes, it is very hard. Because you have a baby that is sick, right? And it is a lot of stress, it is a lot of problems. You have the other things that you are thinking about, more problems. When I am in the hospital, I have everything, they take care of me… but what am I going to do when I go home? And nobody protects you.”

### THEME 6: Recommendation: mitigate harm experienced within and outside of the NICU through peer support and SDH (social drivers of health) resource allocation for families

As a group, participants immensely appreciated the social work team, and found the team to be understaffed and overworked. Their recommendations included more staff and more social services to meet their social and mental health needs. In addition to social work support, families identified the need for mental health providers and a family liaison, who would serve several functions, including as a near peer parent with lived experience in the NICU who can orient families, provide them with knowledge of available local supports and resources including mental health and lactation support, and function as a third party advocate for families.

Focus groups as peer group experiences were described as “therapeutic” themselves, noting peer support, relationship, and community building between parents and staff. During sessions, participants shared both racialized and traumatic experiences and validated each other’s experiences. Participants recommend that parents and staff participate in these experiences given the personal growth that they themselves experienced. One Black parent shared feelings of validation, bidirectional shared empathy, and self-reflection stating, “It was good to know that there was a lot of other families that went through the same things that I went through and that I wasn’t crazy. It was just nice to hear. I felt it very therapeutic and kind of relieving. It felt good to listen to other moms talk about their journeys and what they experienced in the ICN. It took me a while to reflect on them [my emotions]. I had a day to myself the day after to kind of work on them just because it brought up a lot of old trauma I guess from the ICN that I hadn’t worked on yet but it helped. It helped a lot.” The audio diaries and focus group sessions themselves held a significant amount of hope for the future which appeared to be further fueled by productive tough conversations and relationship building between parents and providers during the sessions, despite the challenging topics of discussion. One family support staff member summarized the general feeling of the importance of dialog within the focus group and hope for the future, “There’s radical optimism in that we’re in a different time and we’re not doing it alone”.

## Discussion

In this study we conducted focus groups with Black and Latinx parents and staff in the neonatal intensive care unit to disseminate local racial inequities data, discuss personal experiences of inequities and mechanisms for how they arise, and to generate consensus recommendations. From the focus group and audio diary follow-up data, themes were generated regarding mechanisms by which racial inequities occur in the NICU and recommendations to address them. This study utilizes an innovative hyperlocal parent and staff community driven approach to prioritize community stakeholder perspectives within focus groups to inform recommendations for action in the NICU.

Other studies have highlighted unequal treatment in the NICU from the parent perspective [9, 10, 12, 22]. Special attention has been given to highlighting the voices of Black mothers, who have described their experiences with internalized, interpersonal, institutional, and structural forms of racism in the NICU in two recent studies conducted by Witt et al. and Ajayi et al. [10, 12]. Our study is in alignment with and reinforces several themes from both studies despite recruitment of Black parents from various geographic, political, and neonatal intensive care unit climates in St. Louis, San Francisco, and the U.S. broadly. Black parents were racialized similarly, as uneducated and/or angry and modified their behaviors to avoid retribution and disciplinary action. Although power dynamics were identified in both the present and Witt et al. study, our study additionally offers recommendations for how to redistribute power by treating families as true care partners through orientation, education, and support rather than disciplinary action. In addition, all three studies have highlighted ongoing needs, including increased family, community, and peer support as well as culturally relevant care and resources. More specifically, culturally responsive care and support is operationalized as a more diverse workforce with increased Black representation, recognizing and addressing each family’s unique needs, and improved staff education and training [10, 12]. Coming to a similar conclusion, a study of neonatal experts addressing disparity proposed solutions of racial/cultural concordance, bolstering hospital-based resources, and policy interventions [28].

In addition, the present study also provides the perspectives of Latinx parents in the NICU, a group whose perspectives have been understudied in NICU parent experience literature that is generally lacking diversity [29]. In one recent secondary analysis of two studies recruiting Black and Latinx parents with NICU experience in Boston, St. Louis, and Denver, families reported sources of stress in the individual, hospital, and community levels. Notably, in this study, racism and discrimination were not identified as sources of stress, yet participants identified many factors relevant to racism. For example, hospital stressors including language barriers and poor quality of care, and community level stressors including financial, work, and social stressors were all identified. In our study, Latinx parents largely did not initially explicitly name racism as a factor impacting their care initially, focusing instead on sharing experiences of both high and low quality care. However, after several focus groups, Latinx families did report racism and discrimination. We suspect this information was offered only after trust and community building over several focus groups, although we cannot exclude desirability bias. When Latinx parents were racialized, they were rendered invisible or erased. When poor quality of care was experienced secondary to discrimination, Latinx parents reported neglectful care. This study adds important other contributors to racial and ethnic inequities in health outcomes for Latinx families described in previous literature, that tend to highlight language barriers as the major contributors [9, 30, 31].

This study also highlighted the perspectives and input of staff, who emphasized systems level changes to promote humanism towards staff, such as reduced workload for staff. Parents also recognized the connection between hospital systems showing humanism towards staff to promote staff’s showing of humanism towards parents, both when recalling previous experiences and through perspective-taking during the sessions. Previous literature has shown that addressing systemic issues to improve the workplace experience for healthcare workers may reduce fatigue and levels of burnout [32]. Contributors to burnout include but are not limited to inefficient systems, loss of autonomy, long hours, high intensity of work, frequent overnight call, and loss of autonomy [33, 34]. Burnout symptoms include overwhelming exhaustion, depersonalization, or cynicism towards people and work and may contribute to reduced humanism [32, 34].

Over serial sessions, focus groups also allowed for trust building and power sharing, which highlighted additional insights on solutions in comparison to the one-time interviews with providers and parents in the REJOICE study. While staff and parent audio diaries were primarily glowing reviews of the focus groups, there were also times of tension and confrontation between and within parents and staff groups during the sessions. Perhaps both positive and negative experiences contributed to strong interpersonal connections and the authenticity of the experience for participants.

### Strengths

Our unique methodology provided similar mechanisms and strategies to address local racial inequities to previously described studies, while also providing an intervention. Although we expected community and relationship building, we were surprised to find how therapeutic focus groups were interpreted to be by providers and staff. As an intervention, racial and language concordant peer support groups are currently being studied to address parental stress, anxiety, and sense of belonging for families who have or have had an infant hospitalized in the NICU [35]. Other groups have also studied peer support groups in neonatology for families to support mental health, well-being, and breastfeeding [36–39].

Despite decades of awareness, racial inequities in health outcomes still persist for racially marginalized groups. However, there has been significant progress in strategies to combat racism in the obstetric field, where researchers have generated metrics of racism, discrimination, and person-centered care in healthcare generally, and in obstetrics [40–42]. Most importantly, researchers have moved towards addressing racial inequities by partnering with community organizations and critical community stakeholders to develop initiatives that address obstetric racism, birthing and child health inequities [40, 43–46]. Recognizing the importance of these efforts, the American Academy of Pediatrics and American Committee of Obstetrics and Gynecology have released statements at the importance of addressing racism in maternal and child healthcare [17, 47]. These professional bodies and community focused research such as the present study, all present aligned recommendations to address racial inequities in the NICU. The three main recommendations provided by participants in our study are aligned with previous studies and therefore have potential for effective application across the U.S., and thus should be considered for funding and implementation, and advocated for by healthcare workers, healthcare systems, community members, and policymakers. Future studies should focus on building on the evidence base to implement and evaluate community prioritized interventions to reduce racial inequities in the NICU.

### Limitations

Parents and staff who have had more extreme experiences with racism or discrimination may have been more likely to have volunteered to participate in this study, potentially contributing to selection bias. Additionally, group-think bias is an inherent risk in focus groups, a bias that occurs when participants prescribe to a more dominant view within the group. We cannot exclude the possibility of these biases having occurred in this study.

## Conclusion

Our study adds to efforts to understand the mechanisms by which racial inequities occur and concrete consensus recommendations to address them in the NICU, centering staff and Black and Latinx parents in the process. Aligned with previous research, participants in this study recommended to redistribute power by treating families as true care partners, transform the space and staff to promote humanism and inclusion, and mitigate harm experienced within and outside of the NICU through peer support and social drivers of health resource allocation. Additionally, focus group sessions were well-received as interventions themselves. Future studies should focus on building on the evidence-base to implement and evaluate these interventions to reduce racial inequities in the NICU.

## Supplementary information

Supplementary Appendix. REJOICE Study semi-structured focus group questions

## Data Availability

Individual participant data from focus groups and audio diary transcripts will not be shared. Even when data is de-identified, there is significant risk of identifying participants and their responses, given the small sample size in a small patient and workplace community.
