# Supplementary material for: Parent and staff focus groups to address NICU racial inequities: “There’s radical optimism in that we’re in a different time and we’re not doing it alone”
Source: J Perinatol. 2024 Jul 18;45(3):350–8. doi: 10.1038/s41372-024-02063-6 (PMC11888985; doi:10.1038/s41372-024-02063-6)
Supplement: Supplementary file 1 — Supplementary Appendix. REJOICE Study semi-structured focus group questions [file 41372_2024_2063_MOESM1_ESM.docx]

Supplementary Appendix. REJOICE Study semi-structured focus group questions

*Reviewed REJOICE phase 1 data in presentation format by study researchers.*

Session 1 and 2:

After reporting REJOICE study data for this session:

1. What is your reaction to the data provided today?
   1. If prompts or probes are needed:
      1. What emotions or thoughts come to mind?
2. What is your lived experience and how does it relate to the data presented today?
   1. If prompts or probes are needed:
      1. Is your experience similar to or different from those described today?
3. Why do you think that these racial inequities and experiences occurred in this setting?
   1. If prompts or probes are needed:
      1. What is the root cause or reasons for these racial inequities occuring?
4. How can this institution address these racial inequities? Keep in mind SMART goals (specific, measurable, actionable, relevant, and time-bound)
   1. If prompts or probes are needed: What can the institution do? What can the staff do? What can the leadership do?
5. What are the 3 most important recommendations that you provide to the institution today?

Session 3 and 4:

*Reviewed actionable steps brainstormed in the previous focus group sessions by verbally listing and presenting the previous recommendations.*

1. What key or essential components are most important for each of these interventions?
   1. If prompts or probes are needed:
      1. I.e. For peer support recommendation:
         1. What type of setting is ideal?
         2. What type of person should lead these (family member, social worker, community member, mental health supporter)?
         3. What is their structure
         4. How often should they occur?
         5. Should they be racially or culturally concordant?
      2. For orientation & education recommendation:
         1. What type of support?
         2. What type of support personnel to facilitate it?
         3. What mode of learning? I.e. video or in-person
         4. What other considerations, language, racial/cultural concordance?
2. What will be the potential result/outcome of these interventions?
   1. If prompts or probes are needed:
      1. Would you expect maternal mental health improvement?
      2. Would you expect healthier babies?
      3. Would you expect less complications?
3. What would stop this intervention from happening?
   1. If prompts or probes are needed:
      1. Do you anticipate funding or money would be an issue?
      2. Do you anticipate lack of staff education would be an issue?
4. What would stop you from attending and/or participating in any of these interventions?
   1. If prompts or probes are needed
      1. Child care
      2. Transportation
      3. Perceived safety
5. What resources can we leverage to implement these interventions?
6. Finally, what intervention have we not discussed that you would like to prioritize?
7. What is this single most important intervention, in your opinion?
